# Near-complete genome of medusavirus euryale, putative third species of the genus *Medusavirus*, isolated from the TaeHwaGang river, Ulsan, South Korea

**DOI:** 10.1128/mra.01171-24

**Published:** 2025-02-25

**Authors:** Jiwan Bae, Masaharu Takemura

**Affiliations:** 1 Department of Applied Chemistry, Faculty of Science, Tokyo University of Science, Shinjuku, Tokyo, Japan; 2 Department of Mathematics and Science Education, Graduate School of Science, Tokyo University of Science, Shinjuku, Tokyo, Japan; Katholieke Universiteit Leuven, Leuven, Belgium

**Keywords:** *Medusavirus*, *Mamonoviridae*, *Nucleocytoviricota*, giant virus, Acanthamoeba, South Korea, isolation

## Abstract

We report the isolation and near-complete genome sequencing of medusavirus euryale, a putative third species of the genus *Medusavirus* in the family *Mamonoviridae*, from the TaeHwaGang river in Ulsan, South Korea. Medusavirus euryale has a genome size of approximately 369 kb and contains 446 open reading frames, including two tRNA genes.

## ANNOUNCEMENT

The Genus *Medusavirus* comprises dsDNA viruses that infect *Acanthamoeba castellanii*. To date, only two species of this genus have been isolated (1–3). Here, we isolated a third putative species, medusavirus euryale, from a freshwater sample (50 mL) collected from the TaeHwaGang River in Ulsan, South Korea (35°33′06.6″N, 129°19′26.0″E) on 1 April 2024.

**Fig 1:**
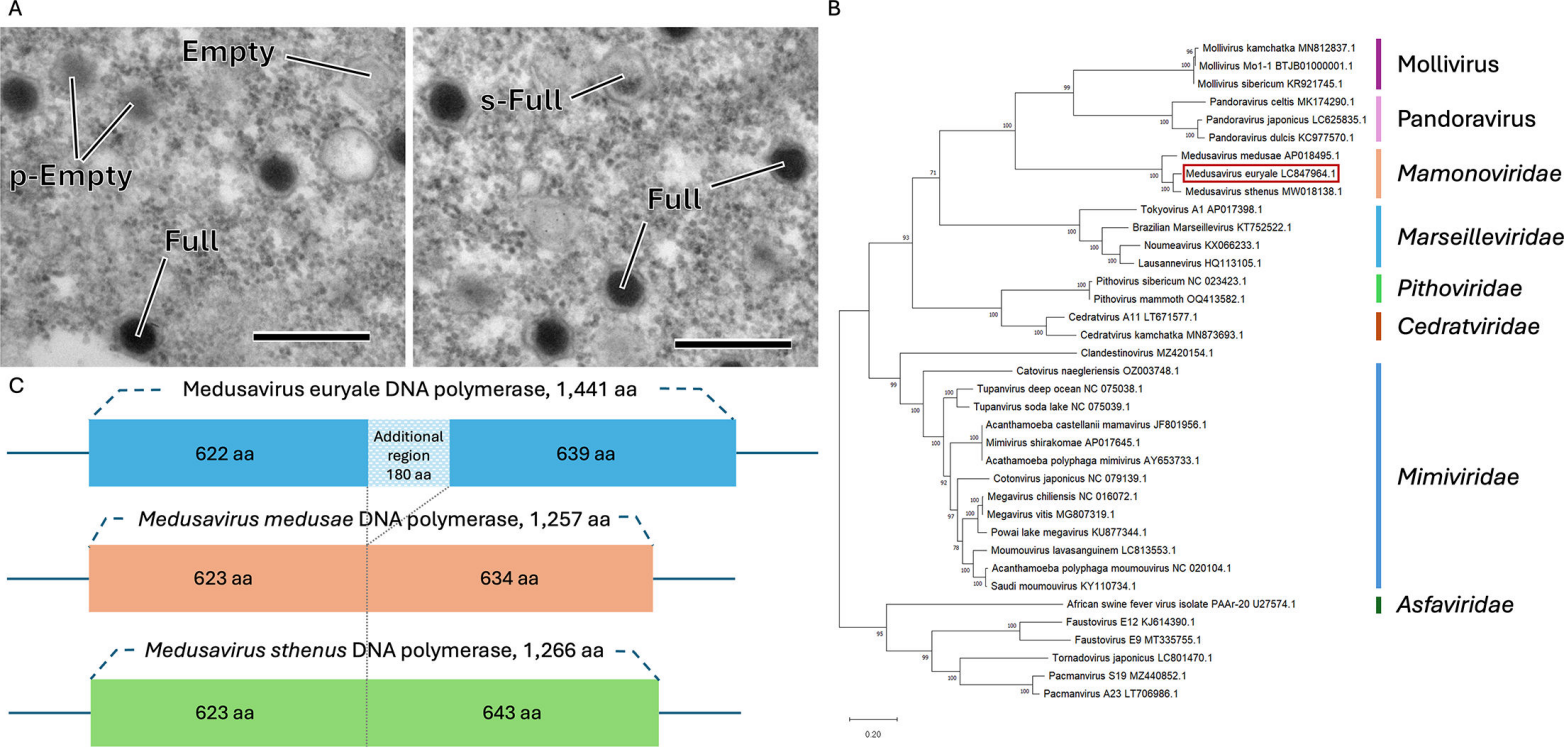
(**A**) Transmission electron microscopy (TEM) image of *Acanthamoeba castellanii* cells infected with medusavirus euryale at 3 days post-infection (dpi). Four types of viral particles are labeled: pseudo-DNA-empty (p-Empty), DNA-empty (Empty), semi-DNA-full (s-Full), and DNA-full (Full). These types of virions have already been reported in *Medusavirus medusae* (6). Scale bar  =  400  nm. (**B**) Phylogenetic tree of B-family DNA polymerase nucleotide sequences. The analysis was performed using MEGA 11 software (7), with alignment generated by the MUSCLE algorithm and the maximum likelihood method applied for tree reconstruction with 1,000 replicates (8). Medusavirus euryale is highlighted in the red box. (**C**) Comparison of amino acid sequences of DNA polymerases across medusavirus species. The medusavirus euryale DNA polymerase has an additional region not shown in *M. medusae* and *M. sthenus*.

The water sample was mixed and co-cultured with *A. castellanii* strain Neff. The supernatant from the well showing cytopathic effects was transferred to fresh cells and cultured, followed by cloning of virus as described previously (1, 4, 5). Virus-infected *Acanthamoeba* cells were visualized via transmission electron microscopy (TEM) (Fig. 1A). Although the specific morphology of this virus has not been fully investigated, it is expected to exhibit an icosahedral structure similar to previously identified medusavirus species (1). Four distinct viral particle types were observed in infected cells via TEM (Fig. 1A), consistent with previous findings (6).

Genomic DNA was extracted from the supernatants of infected cells using the phenol/chloroform method (9, 10), with minor modifications. Samples were resuspended in 100 µL of TES buffer (50 mM Tris-HCl pH 7.6, 25% sucrose, 20 mM EDTA, 0.5 mg/mL lysozyme, and 1 mg/mL RNase A). After 30 min of incubation at 37°C, Proteinase K (0.17 mg/mL) and SDS (0.43%) were added, and the mixture was further incubated for 30 min at 37°C. The lysate obtained was then treated with phenol/chloroform, followed by ethanol precipitation. For whole-genome sequencing, a DNA library was prepared using the TruSeq Nano DNA Library (350) kit (Illumina, Inc.). Sequencing was performed on the NovaSeq X Plus platform (Illumina, Inc.), yielding 35,377,848 reads (5,342,055,048 total read bases). Raw sequence data were quality-checked using FastQC (v0.11.7). The reads were assembled using SPAdes (v3.15.0), producing a single 369,026 bp contig (coverage 99.92%, depth of coverage 9,592×). The assembled genome was validated using self-mapping strategy (BWA v0.7.17-r1188) (11) and BLASTn (v2.14.0). Open reading frames (ORFs) were predicted using Prokka software (v1.14.6) (12). The coding sequences (CDSs) were annotated using the NCBI BLASTp database (v2.14.0) (13). Default parameters were used in all software. The G + C content in the genome was 63.1%. A total of 446 ORFs were identified, including 444 CDSs and two tRNA genes. Of the 446 ORFs, 399 matched medusavirus sequences revealed by BLASTp (13), while 16 were homologous to other viruses (such as pandoravirus and mollivirus), *A. castellanii*, or other organisms. Molecular phylogenetic analysis indicated that this virus belongs to the genus *Medusavirus* (Fig. 1B). ANI (average nucleotide indentity) of this virus was 79.89% with *M. medusae* and 87.79% with *M. sthenus* (14). While medusavirus euryale encodes all core histones, with H3 and H4 fused into a single gene as in *M. sthenus* (2), we did not find evidence of linker histone H1. We found that the medusavirus euryale B family DNA polymerase possessed an 180 aa insertion compared with *M. medusae* and *M. sthenus* (Fig. 1C).

## Data Availability

The sequence data are available in GenBank (LC847964.1) under BioProject PRJDB18840 and SAMD00820991, and raw reads were obtained from the Sequence Read Archive (DRR618977).
